# Supplementary material for: Janus 3D printed dynamic scaffolds for nanovibration-driven bone regeneration
Source: Nat Commun. 2021 Feb 15;12:1031. doi: 10.1038/s41467-021-21325-x (PMC7884435; doi:10.1038/s41467-021-21325-x)
Supplement: Supplementary file 1 — Supplementary Information [file 41467_2021_21325_MOESM1_ESM.pdf]

Janus 3D printed dynamic scaffolds for nanovibration-driven bone regeneration

Sandra Camarero-Espinosa[1,2,3] and Lorenzo Moroni[1]

[1]MERLN Institute for Technology-inspired Regenerative Medicine, Complex Tissue Regeneration Department, Maastricht University, P.O. Box 616, 6200 MD Maastricht, The Netherlands

[2] POLYMAT, University of the Basque Country UPV/EHU, Avenida Tolosa 72, Donostia / San Sebastián 20018, Gipuzkoa, Spain.

[3]IKERBASQUE, Basque Foundation for Science, Bilbao, Spain

Supplementary Information

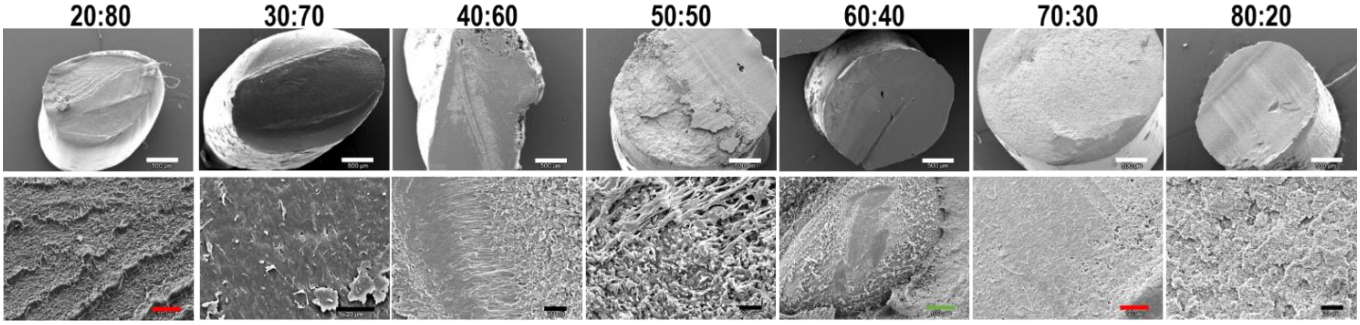

Supplementary Figure 1. Scanning Electron Microscopy (SEM) images of as-extruded filaments of PLA:PCL blends where an initial phase-segregation is observed. PLA concentration increases from left to right. White scale bars are 500 µm, green are 100 µm, red are 50 µm and black are 20 µm. The experiment was performed with three replicates with similar results.

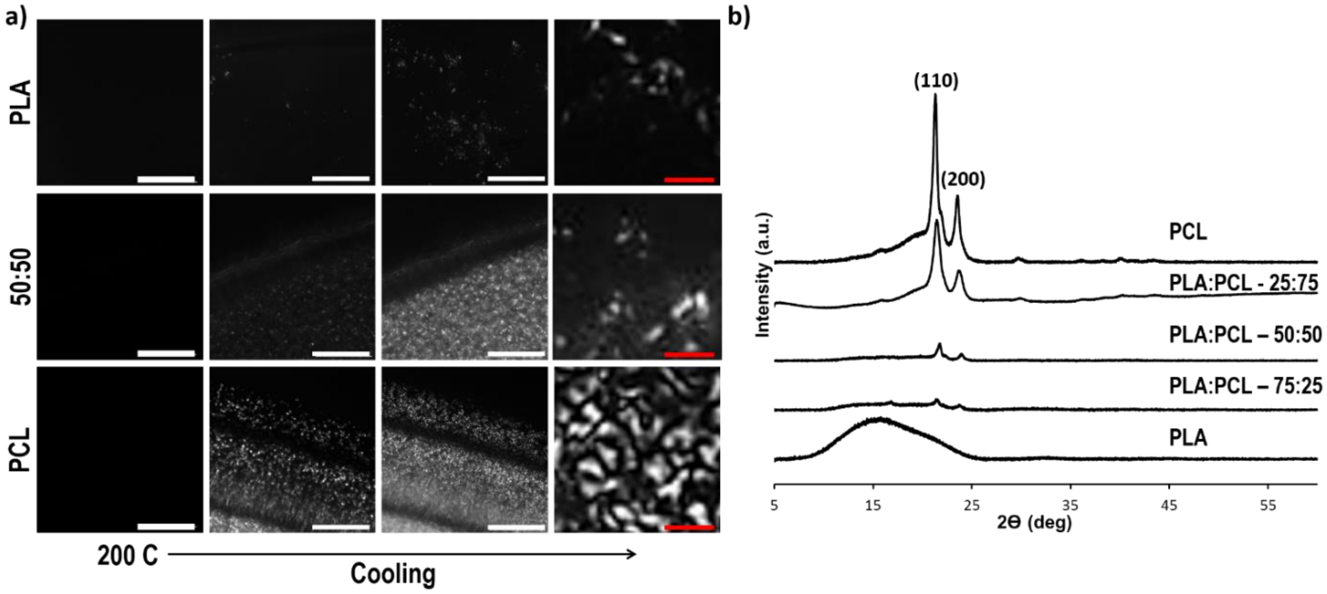

Supplementary Figure 2. a) Polarized optical microscopy images of PLA (top), 50:50 PLA:PCL blend (middle) and PCL (bottom) upon cooling from 200°C melts showing the crystallization of PCL in the neat state and mixed with PLA. Scale bars are 100µm, red scale bars are 10 µm. b) X-ray diffraction spectra of PLA, PCL and PLA:PCL blends at 75:25, 50:50 and 25:75 ratios showing a progressive increase on the intensity of the crystallization peaks of PCL (110 and 200) and a characteristic amorphous hallo of PLA. The experiment was performed with five replicates with similar results.

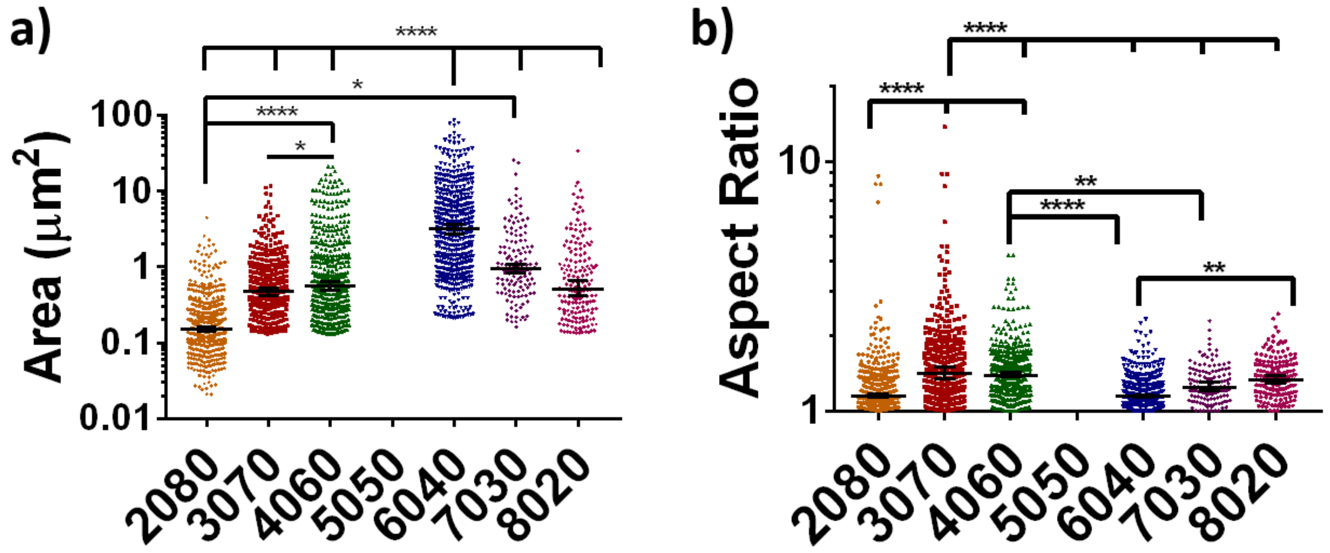

Supplementary Figure 3. Analysis of the area (a) and aspect ratio (b) of the phase segregated particles on the cross-sectional direction for PLA:PCL ratios of 20:80, 30:70, 40:60, 60:40, 70:30 and 80:20 (n = 654, 395, 528, 0, 562, 136 and 175 particles). Sample 50:50 is not included in the analysis. Bars show median and 95% CI. Statistical significance was calculated from 1-way ANOVA with Tukey`s multiple comparison test. (****) p<0.0001, (***) p<0.001, (**) p<0.01 and (*) p<0.1. Source data and exact p values are provided in the source data file.

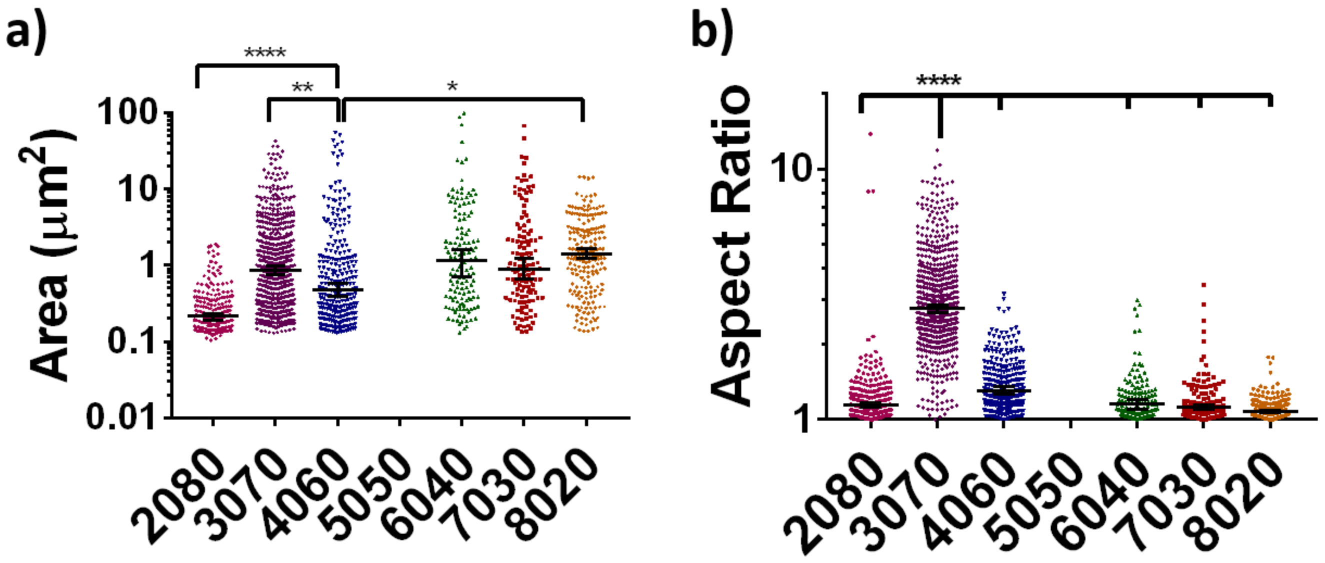

Supplementary Figure 4. Analysis of the area (a) and aspect ratio (b) of the phase segregated particles on the longitudinal direction of the scaffold fibers for PLA:PCL ratios of 20:80, 30:70, 40:60, 60:40, 70:30 and 80:20 (n = 230, 734, 295, 0, 136, 128 and 208 particles). Sample 50:50 is not included in the analysis. Bars show median and 95% CI. Statistical significance was calculated from 1-way ANOVA. (****) $p<0.0001$, (***) $p<0.001$, (**) $p<0.01$ and (*) $p<0.1$. Source data and exact p values are provided in the source data file.

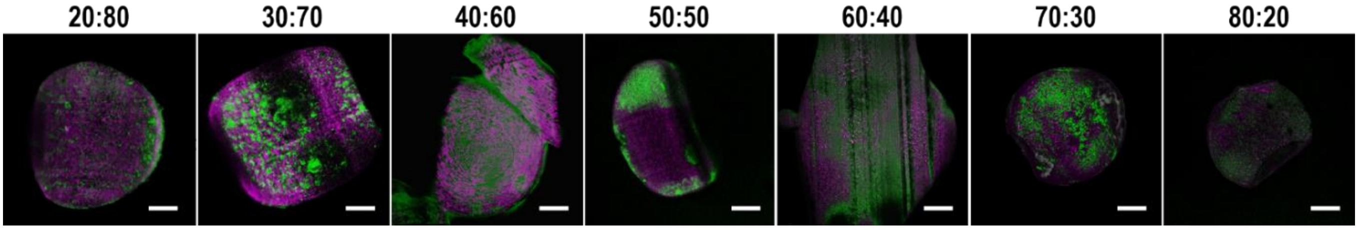

Supplementary Figure 5. Phase segregation of PLA:PCL blends labelled with FITC (488, green) and Rhodamine B (580, pink), respectively, on the cross-section of additive manufactured fibers as visualized by Light Scanning Microscopy (LSM). PLA concentration increases from left to right images. At 50:50 PLA:PCL composition a clear Janus phase segregation is observed. Scale bars are 100 µm. The experiment was performed with three independent replicates with similar results.

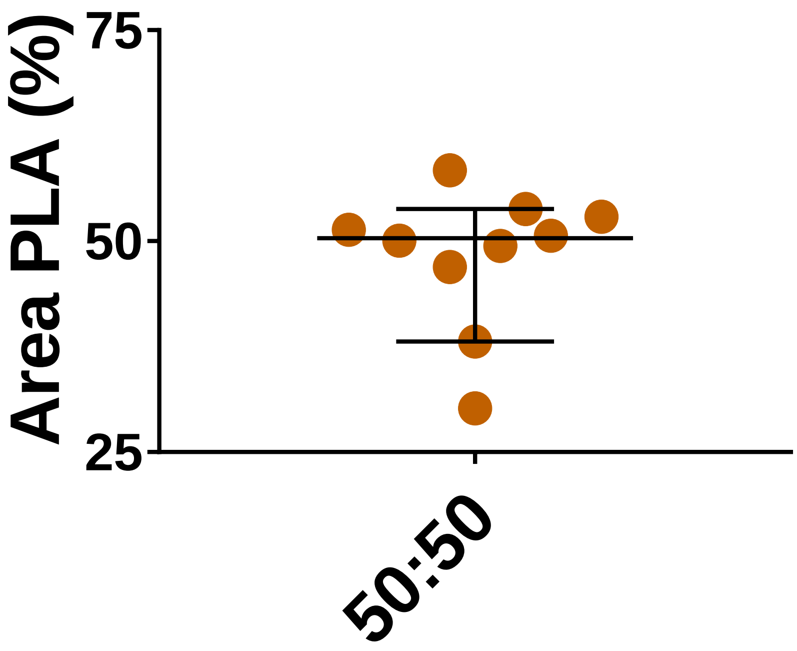

Supplementary Figure 6. Analysis of the % of the total cross-sectional area occupied by the PLA phase on scaffold fibers for a PLA:PCL ratio of 50:50. Bars show median and 95% CI. Brown dots are individual data points. n = 10 images over 5 independent experiments. Source data and exact p values are provided in the source data file.

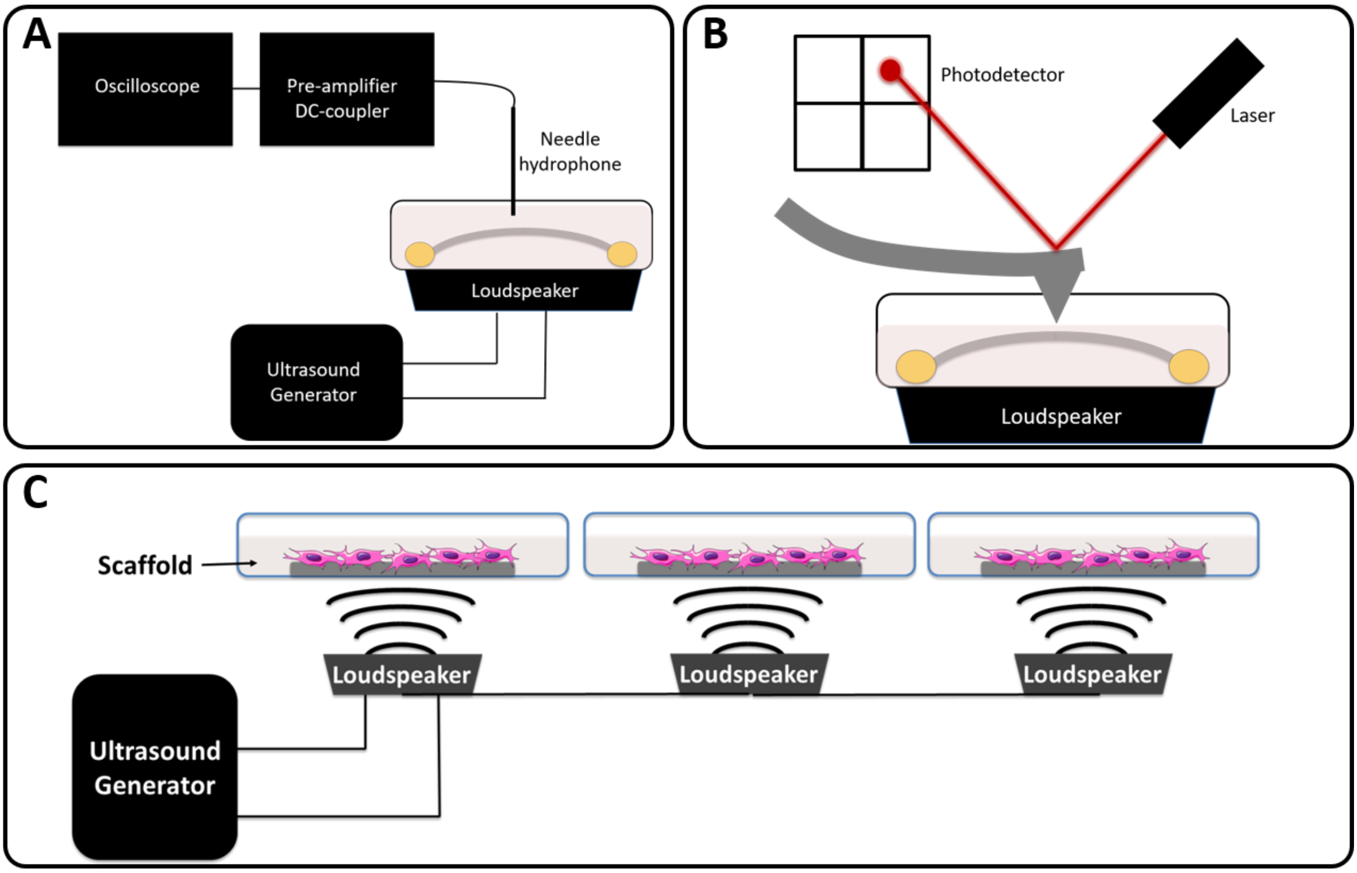

Supplementary Figure 7. Schematic representation of the ultrasound (A) detection set-up, measurement of deflection (B) and cell (C) stimulation.

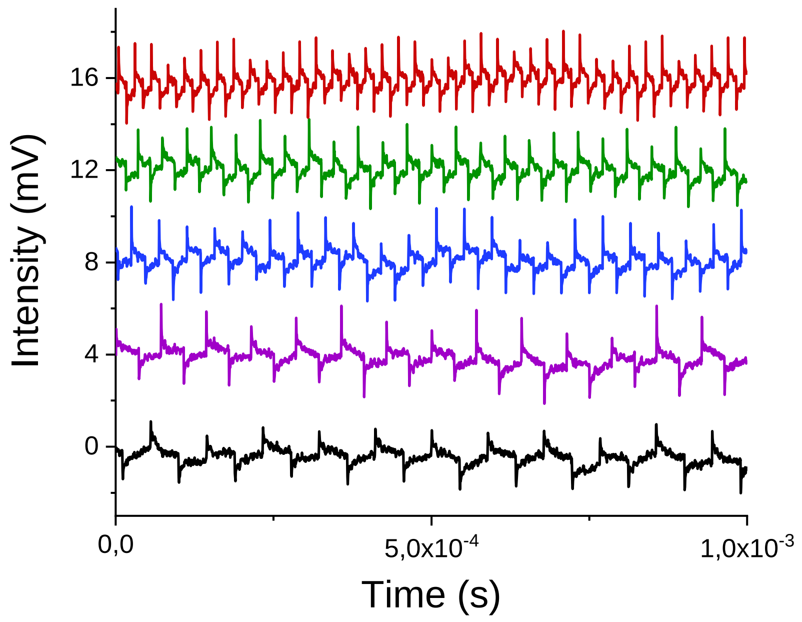

Supplementary Figure 8. Representative ultrasound waves generated to stimulate the scaffolds as detected with a needle hydrophone in liquid media after crossing the same polystyrene layer (Petri-dish) used in experiments with scaffolds, showing a decrease of the frequency from top to bottom.

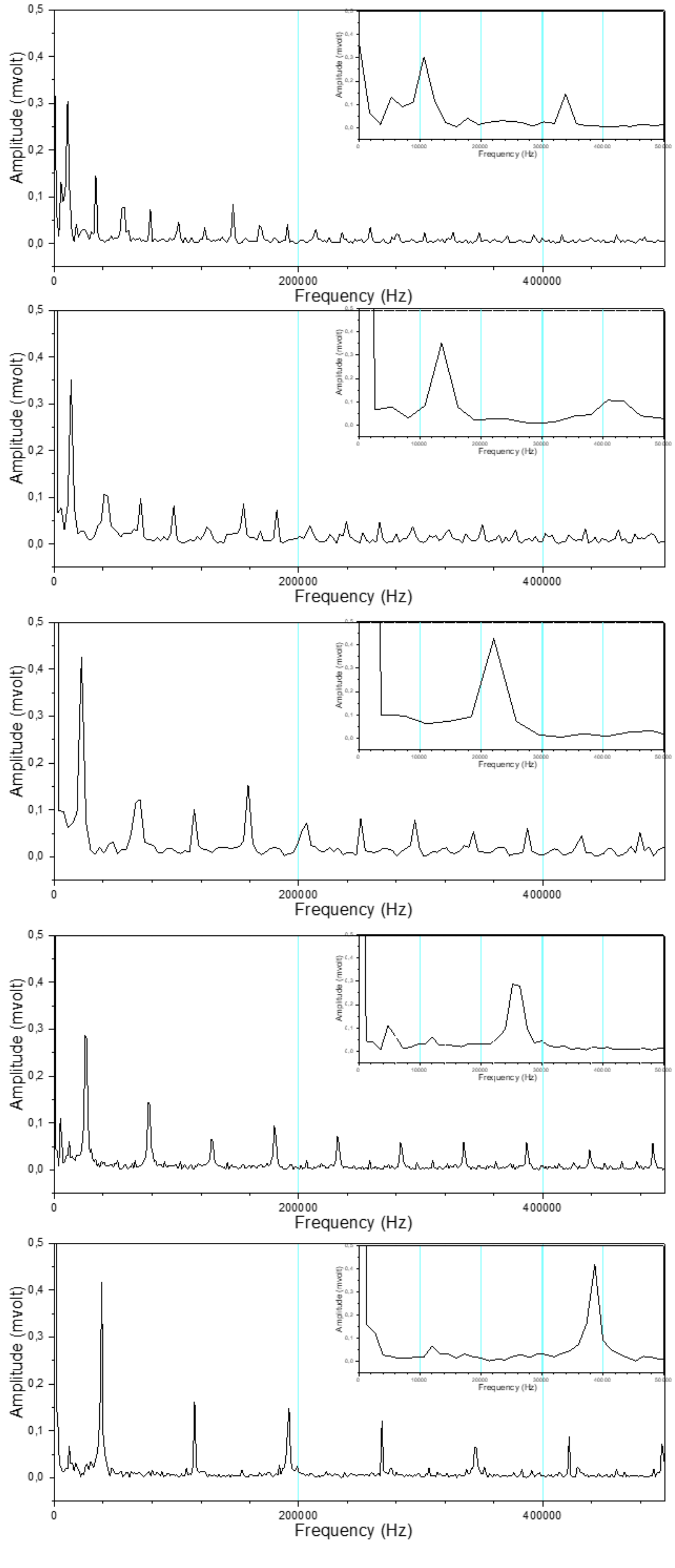

Supplementary Figure 9. Fast Fourier Transform (FFT) of the ultrasound waves used to stimulate the scaffolds showing (from top to bottom) an increase on the frequency.

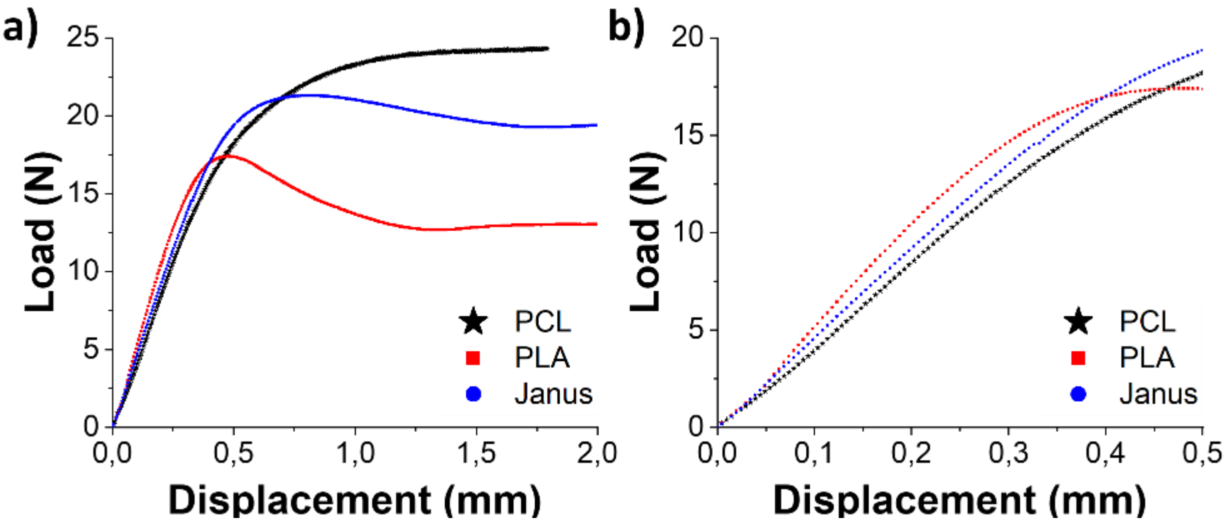

Supplementary Figure 10. Representative load-displacement traces of PCL, PLA and Janus scaffolds measured under 3-point bending with a 40N load cell at a 0,01mm/s deformation rate. Measurements were done in triplicate samples.

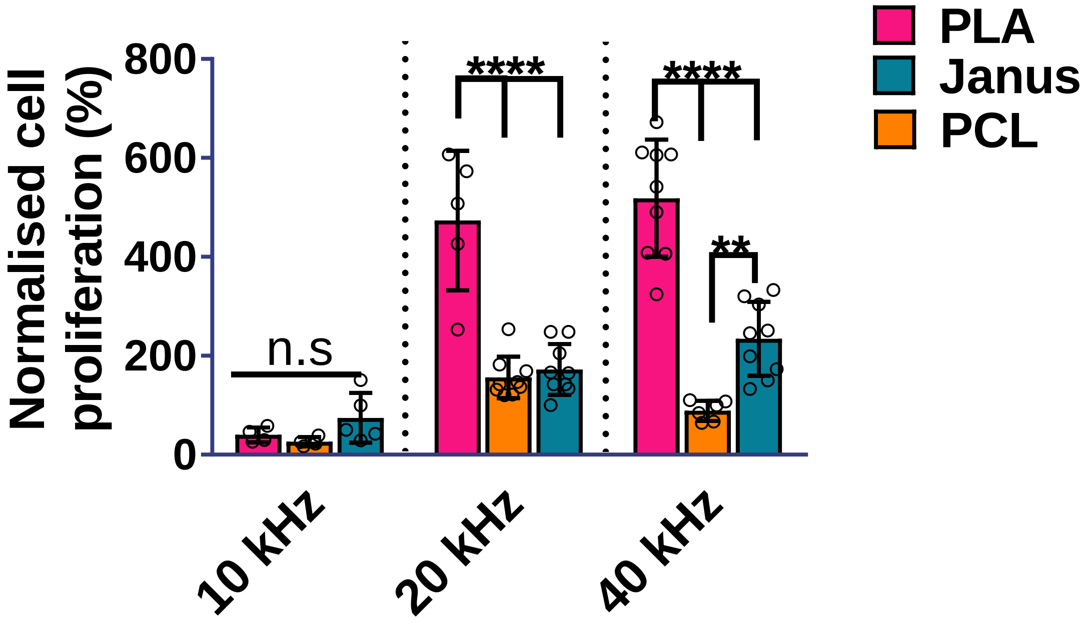

Supplementary Figure 11. hBMSC number at 10, 20 or 40 kHz stimulation as normalized to the cell number at 0 kHz (normalized cell proliferation, %) after 7 days of culture on PLA, Janus and PCL scaffolds. Data is presented as mean ± standard deviation. Statistical significance was calculated by two-way ANOVA with Tukey`s multiple comparison test; (****) $p<0.0001$, (***) $p<0.001$, (**) $p<0.01$ and (*) $p<0.1$. For stimulation at 10, 20 and 40 kHz, n = 4, 5 and 9 for PLA; n = 5, 9 and 6 for PCL and n = 5, 9 and 9 for Janus, respectively. Data is shown as means ± SEM. Black circles are individual data points. Source data and exact p values are provided in the source data file.

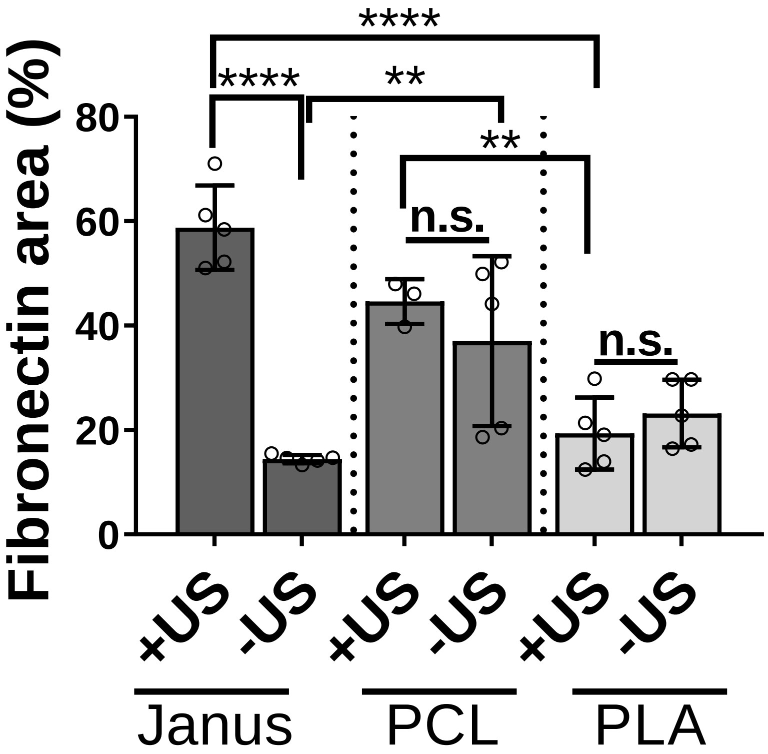

Supplementary Figure 12. Quantification of the area covered with fibronectin on ultrasound stimulated (+US) and non-stimulated Janus, PCL and PLA scaffolds (-US) after 2 weeks of BMSC culture. The data is shown as mean ± standard deviation. Statistical significance was calculated by two-way ANOVA with Tukey`s multiple comparison test between groups and uncorrected Fisher`s LSD test between conditions of a same group (+/- US); (****) $p<0.0001$, (***) $p<0.001$, (**) $p<0.01$ and (*) $p<0.1$ n = 5 images analyzed over 3 independent experiments, except PCL +US where n = 3. Black circles represent individual data points. Source data and exact p values are provided in the source data file.

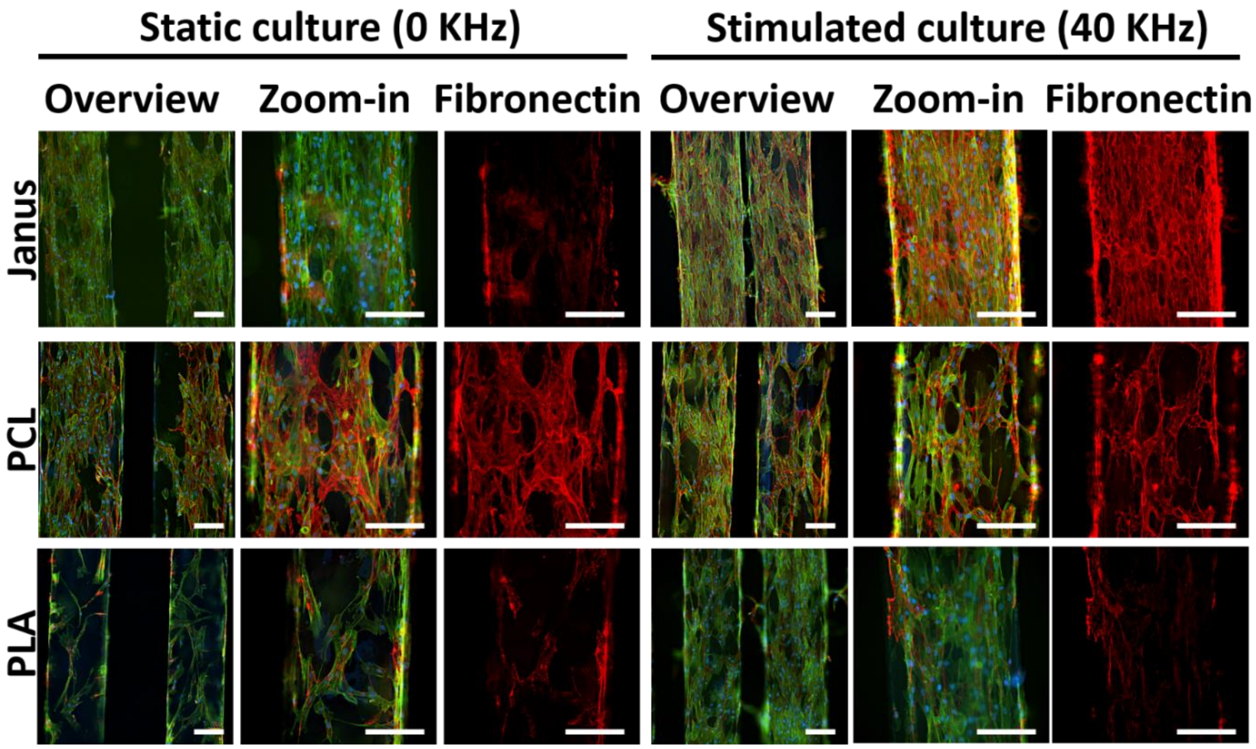

Supplementary Figure 13. Fluorescent light microscopy images of BMSCs cultured for 1 week on Janus, PCL and PLA scaffolds and stimulated 30 minutes daily at 40kHz showing an increased fibronectin deposition on Janus scaffolds. Cells were stained for F-actin (green), DNA (blue) and fibronectin (red). Scale bar is 200 µm. The experiment was performed with three independent biological samples with similar results.

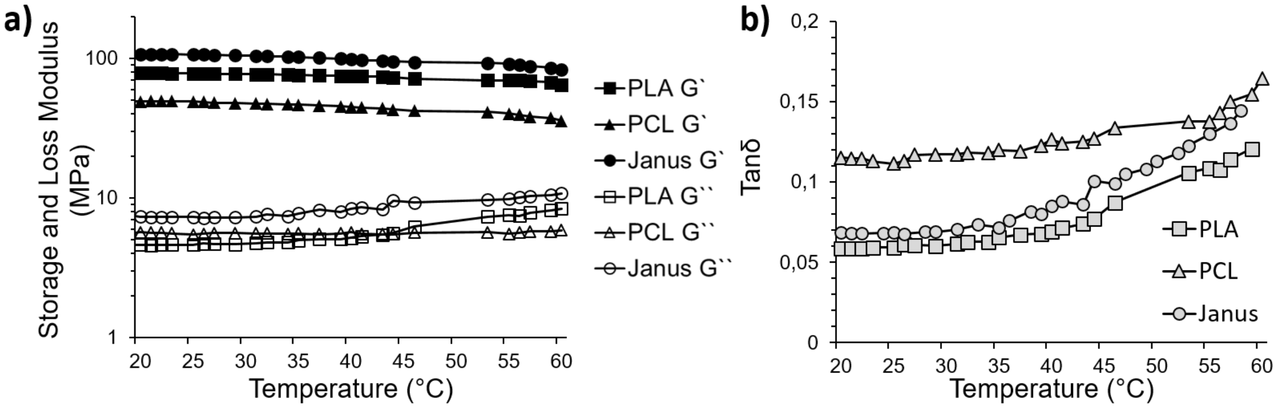

Supplementary Figure 14. Representative dynamic mechanical thermal analysis curves showing the (a) storage (G`) and loss (G``) moduli and (b) the resulting tangent δ (tan) of PLA, PCL and Janus 3D printed fibers at a frequency of 0.1 Hz.

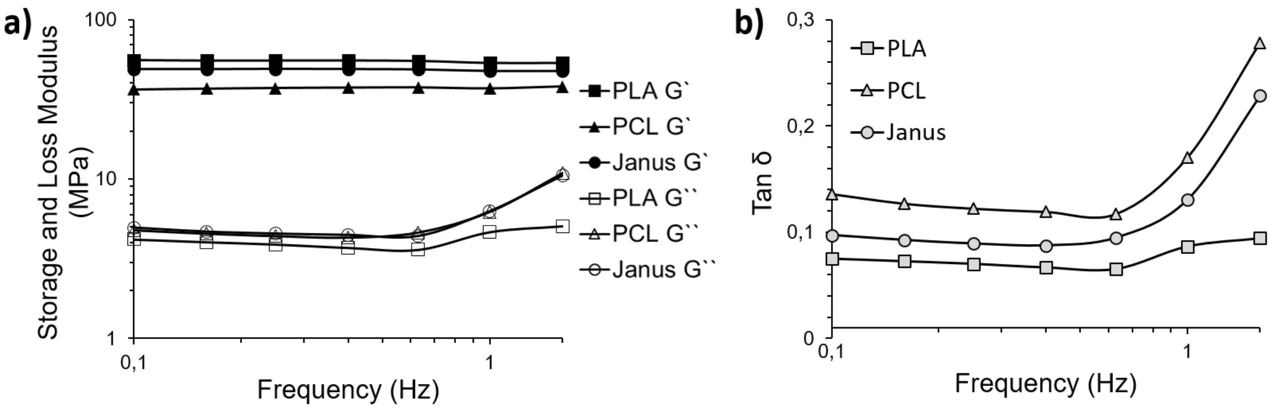

Supplementary Figure 15. Representative dynamic mechanical analysis curves showing the (a) storage (G`) and loss (G``) moduli and (b) the resulting tangent δ (tan) of PLA, PCL and Janus 3D printed fibers at 37 °C and over a one decade frequency sweep.

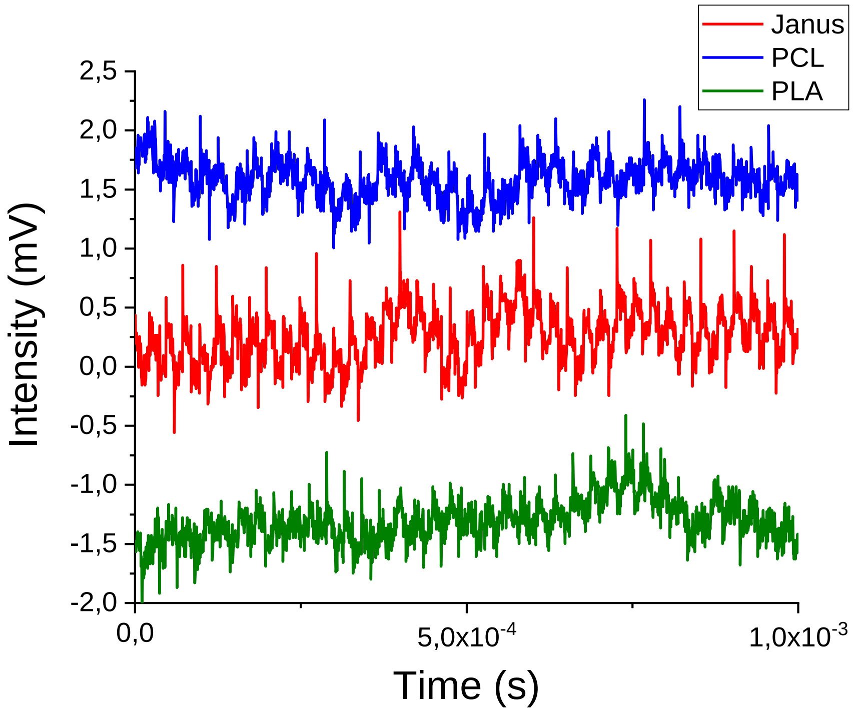

Supplementary Figure 16. Representative ultrasound waves transmitted after crossing a polystyrene layer (Petri-dish) and the different scaffold compositions (Janus, PCL and PLA), measured with a needle hydrophone in liquid media. Scaffolds were excited with an ultrasound wave of 38 kHz.

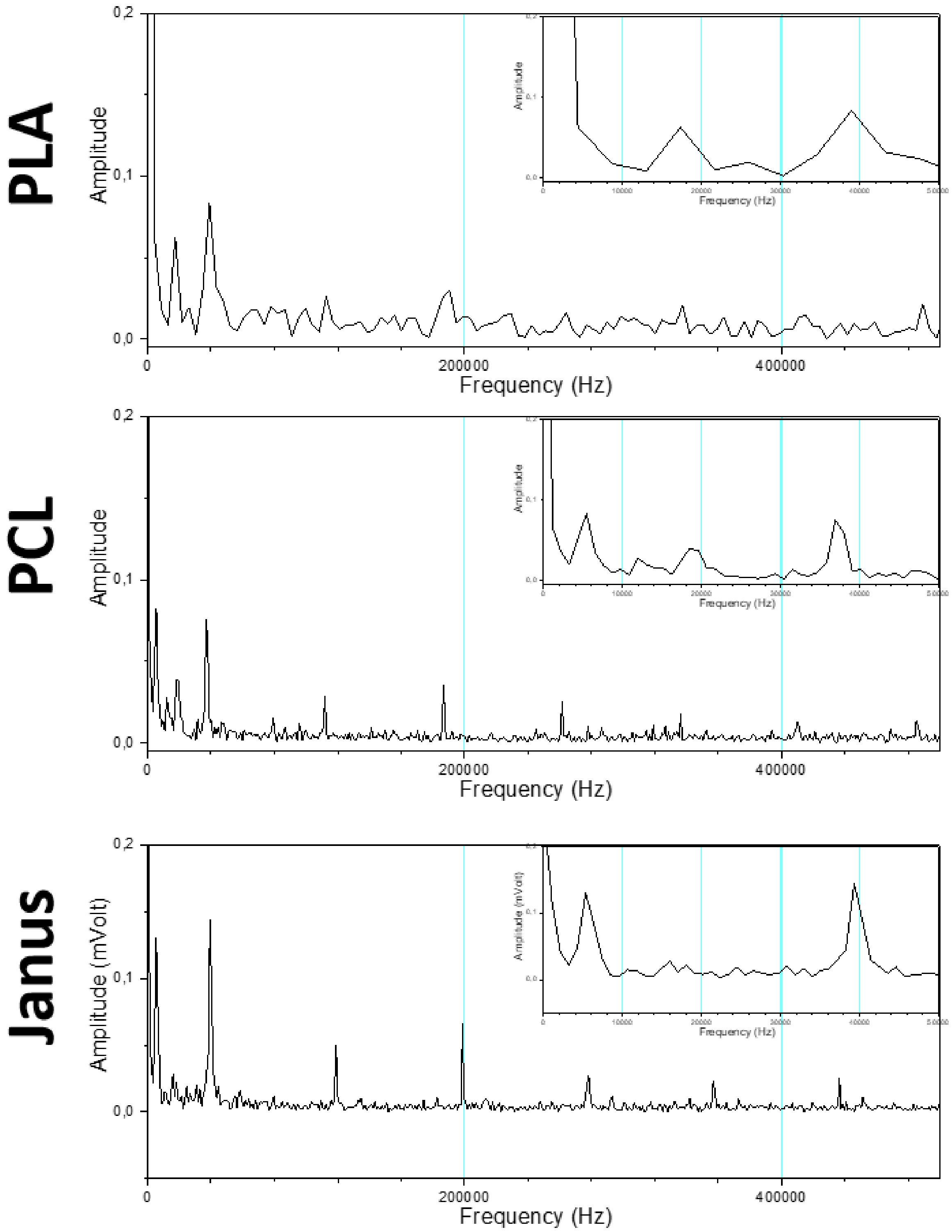

Supplementary Figure 17. Fast Fourier Transform (FFT) of the ultrasound transmitted through the scaffolds showing (from top to bottom) an increase on the frequency of the first peak corresponding to the scaffolds transmitted wave, and a secondary one corresponding to the emitted wave (38 kHz).

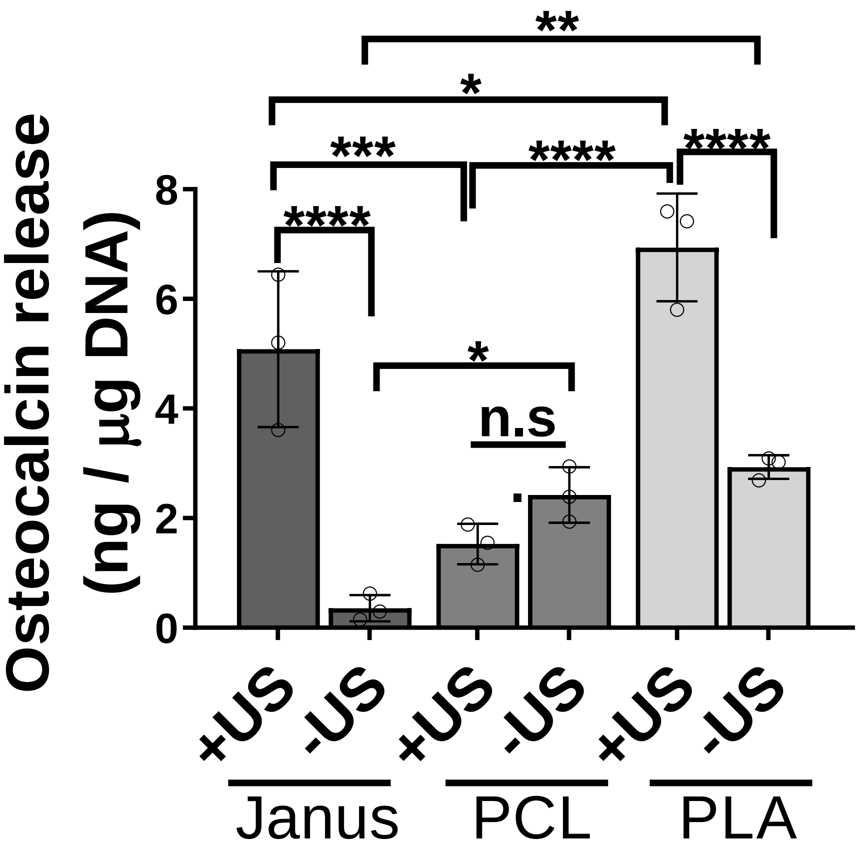

Supplementary Figure 18. Osteocalcin release normalized to total DNA content after 3 weeks of osteogenic differentiation of BMSC cultured on Janus, PCL and PLA scaffolds under stimulated (+US) and static (-US) conditions, showing an ultrasound dependent release for cells cultured on Janus and PLA scaffolds that was higher for Janus than PCL scaffolds. Data is shown as mean ± standard deviation. Statistical significance was calculated by two-way ANOVA with Tukey`s multiple comparison test between groups and uncorrected Fisher`s LSD test between conditions of a same group (+/- US); (****) $p<0.0001$, (***) $p<0.001$, (**) $p<0.01$ and (*) $p<0.1$. n = 3 biologically independent samples. Black circles represent individual data points. Source data and exact p values are provided in the source data file.

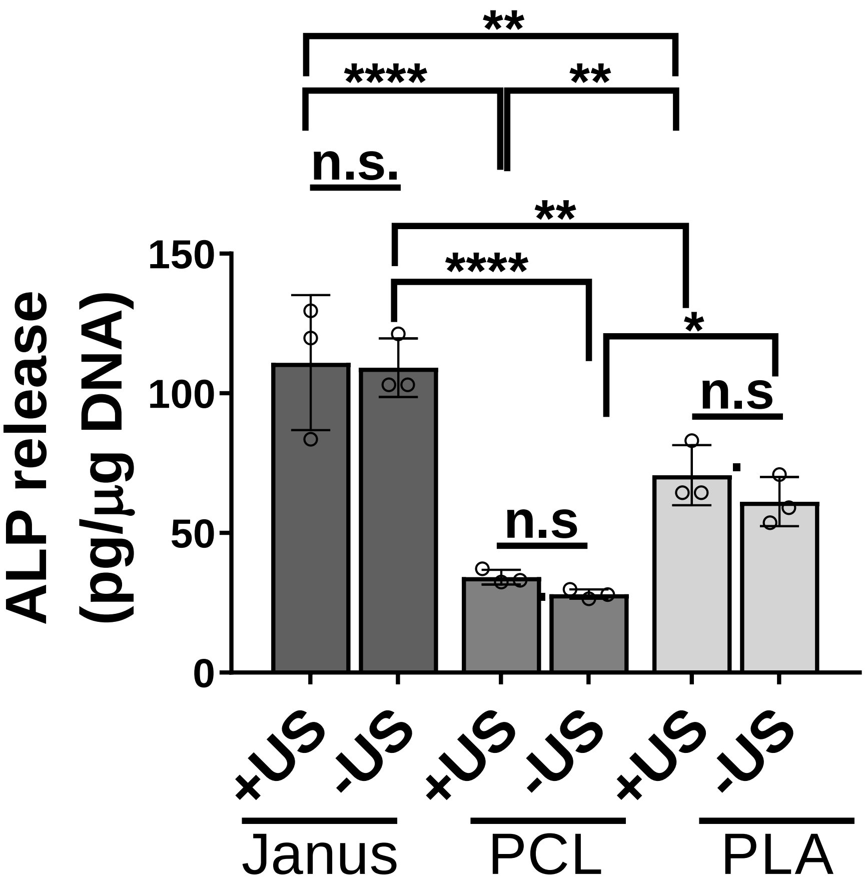

Supplementary Figure 19. Alkaline phosphatase release normalized to total DNA content after 3 weeks of osteogenic differentiation of BMSC cultured on Janus, PCL and PLA scaffolds under stimulated (+US) and static (-US) conditions, showing an ultrasound-independent release that is maximum for cells cultured on Janus scaffolds. Data is shown as mean ± standard deviation. Statistical significance was calculated by two-way ANOVA with Tukey`s multiple comparison test between groups and uncorrected Fisher`s LSD test between conditions of a same group (+/- US); (****) p<0.0001, (***) p<0.001, (**) p<0.01 and (*) p<0.1. n = 3 biologically independent samples. Black circles represent individual data points. Source data and exact p values are provided in the source data file.

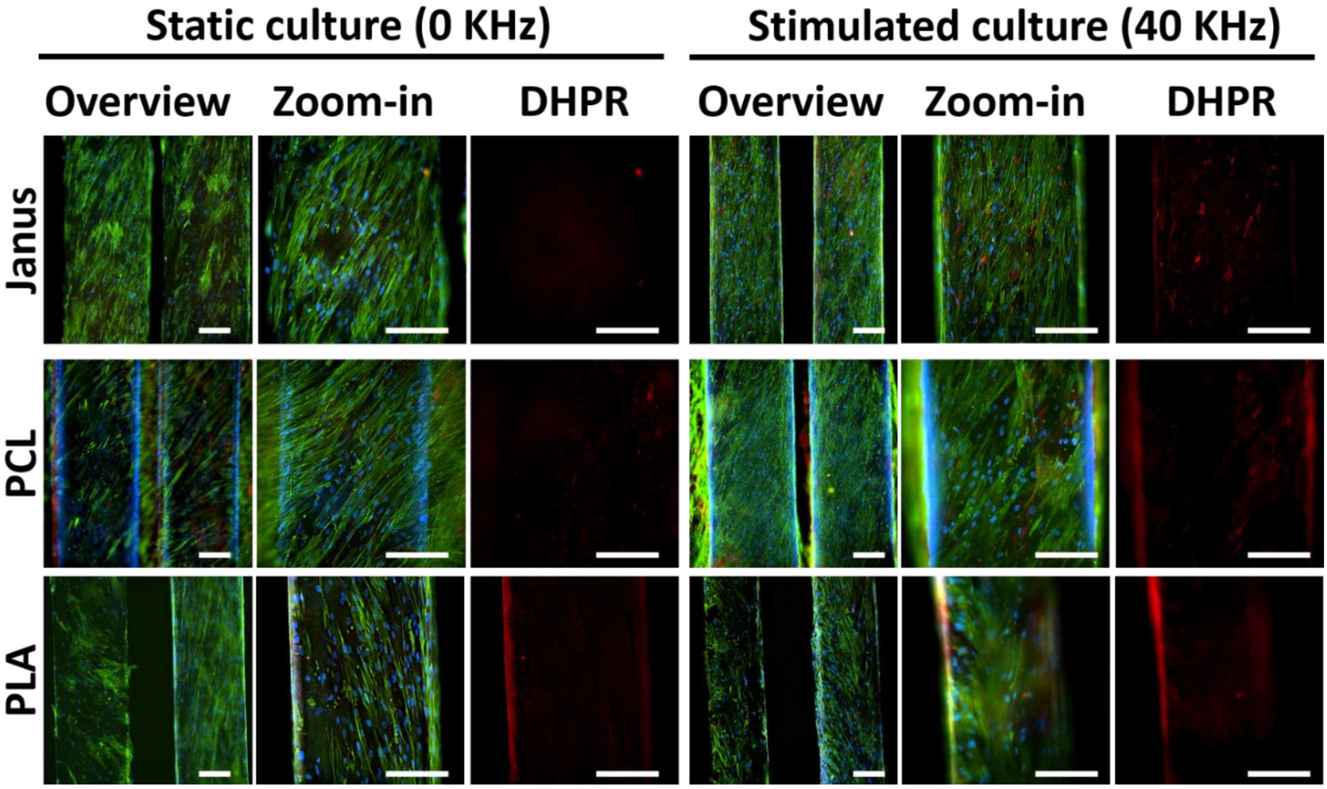

Supplementary Figure 20. Fluorescent light microscopy images of BMSCs cultured for 3 weeks on Janus, PCL and PLA scaffolds in osteogenic media and stimulated 30 minutes daily at 40kHz, showing the expression of dihydropyridine receptor (DHPR, a voltage gated Ca^{2+} ion channel) on Janus scaffolds. Cells were stained for F-actin (green), DNA (blue) and DHPR (red). Scale bar is 200 µm. The experiment was performed with three independent biological samples with similar results.

Supplementary Table 1. Measured frequency and amplitude of the different generated ultrasound waves. Set-ups 2, 6 and 10 were used for cell work and are referred as 10, 20 and 40 kHz. Data corresponds to the representative FFTs shown in Figure S7.

Set-up	Frequency (kHz)	Intensity (mV)
**2**	11,7	0,3
**4**	13,6	0,3
**6**	22,2	0,3
**8**	25,5	0,3
**10**	38,7	0,4

Supplementary Table 2. Flexural modulus (MPa) calculated for the different scaffolds compositions; PLA, PCL and Janus. Data is shown as mean ± SD, n = 3.

Material	Flexural modulus (MPa)
**PLA**	2326 ± 36
**PCL**	303 ± 70
**Janus**	759 ± 62

Supplementary Table 3. Measured frequencies and intensities of the ultrasound waves transmitted by the scaffolds. Data corresponds to the representative FFTs shown in Figure S10.

Material	Frequency	Intensity (mV)
**PCL**	5,4	0,08
	36,8	0,07
**PLA**	17,3	0,06
	38,9	0,08
**Janus**	15,4	0,05
	38,5	0,1
