# Supplementary material for: Janus 3D printed dynamic scaffolds for nanovibration-driven bone regeneration
Source: Nat Commun. 2021 Feb 15;12:1031. doi: 10.1038/s41467-021-21325-x (PMC7884435; doi:10.1038/s41467-021-21325-x)
Supplement: Supplementary file 3 — Reporting Summary [file 41467_2021_21325_MOESM3_ESM.pdf]

nature research

Corresponding author(s): Prof. Lorenzo Moroni

Last updated by author(s): Dec 20, 2020

Reporting Summary

Nature Research wishes to improve the reproducibility of the work that we publish. This form provides structure for consistency and transparency in reporting. For further information on Nature Research policies, see our Editorial Policies and the Editorial Policy Checklist.

Statistics

For all statistical analyses, confirm that the following items are present in the figure legend, table legend, main text, or Methods section.

n/a	Confirmed	
☐	☒	The exact sample size (*n*) for each experimental group/condition, given as a discrete number and unit of measurement
☐	☒	A statement on whether measurements were taken from distinct samples or whether the same sample was measured repeatedly
☐	☒	The statistical test(s) used AND whether they are one- or two-sided<br>*Only common tests should be described solely by name; describe more complex techniques in the Methods section.*
☐	☒	A description of all covariates tested
☐	☒	A description of any assumptions or corrections, such as tests of normality and adjustment for multiple comparisons
☐	☒	A full description of the statistical parameters including central tendency (e.g. means) or other basic estimates (e.g. regression coefficient) AND variation (e.g. standard deviation) or associated estimates of uncertainty (e.g. confidence intervals)
☐	☒	For null hypothesis testing, the test statistic (e.g. *F*, *t*, *r*) with confidence intervals, effect sizes, degrees of freedom and *P* value noted<br>*Give P values as exact values whenever suitable.*
☒	☐	For Bayesian analysis, information on the choice of priors and Markov chain Monte Carlo settings
☒	☐	For hierarchical and complex designs, identification of the appropriate level for tests and full reporting of outcomes
☒	☐	Estimates of effect sizes (e.g. Cohen's *d*, Pearson's *r*), indicating how they were calculated

Our web collection on statistics for biologists contains articles on many of the points above.

Software and code

Policy information about availability of computer code

Data collection	COMSOL Multiphysics 5.4
Data analysis	CellProfiler 3.1.9, GraphPad Prism 7, Origin 2018 95E, WinTest DMA 7.1, FiJi 2.0.0-pre-8

For manuscripts utilizing custom algorithms or software that are central to the research but not yet described in published literature, software must be made available to editors and reviewers. We strongly encourage code deposition in a community repository (e.g. GitHub). See the Nature Research guidelines for submitting code & software for further information.

Data

Policy information about availability of data

All manuscripts must include a data availability statement. This statement should provide the following information, where applicable:

- Accession codes, unique identifiers, or web links for publicly available datasets
- A list of figures that have associated raw data
- A description of any restrictions on data availability

Data underlying the figures can be found in the Supplementary Data Source. All raw data is stored on https://datahub.io/ and is available from the authors upon reasonable request.

Field-specific reporting

Life sciences study design

All studies must disclose on these points even when the disclosure is negative.

Sample size	No sample size calculation was performed. Data analysis was n=3 with technical and biological triplicates in all cases. n = 3 was chosen as it is the commonly accepted cumulative frequency in biological in vitro research.
Data exclusions	No data was excluded from analysis
Replication	All, material preparation, processing and characterization were performed a minimum of 3 times. Biological studies were performed 3 times with technical and biological triplicates all showing the same trend.
Randomization	Printed objects of a given material were assigned to different treatment groups randomly.
Blinding	Investigators were not blinded to group allocation since differences between groups were clear to the researchers by naked eye. Randomization was not relevant to our study as quantitative data was obtained. Qualitative data is reported without further treatment.

Reporting for specific materials, systems and methods

We require information from authors about some types of materials, experimental systems and methods used in many studies. Here, indicate whether each material, system or method listed is relevant to your study. If you are not sure if a list item applies to your research, read the appropriate section before selecting a response.

Materials & experimental systems

n/a	Involved in the study	
☐	☒	Antibodies
☐	☒	Eukaryotic cell lines
☒	☐	Palaeontology and archaeology
☒	☐	Animals and other organisms
☒	☐	Human research participants
☒	☐	Clinical data
☒	☐	Dual use research of concern

Methods

n/a	Involved in the study	
☒	☐	ChIP-seq
☒	☐	Flow cytometry
☒	☐	MRI-based neuroimaging

Antibodies

Antibodies used	rabbit anti-fibronectin (Abcam ab2413), mouse anti-collagen I (Abcam ab90395), mouse anti-DHPR alpha 2 subunit(Abcam ab2864), rabbit anti-Ryanodine (Abcam ab219798), Alexa Fluor 488-phalloidin, Alexa Fluor 568-phalloidin
Validation	Abcam ab2413: IHC-P: Manufacturer validation; Human kidney tissue. WB: Human colon lysate; HepG2 and NIH/3T3 cell lysate. ICC/IF: HeLa cells. 385 references<br>Abcam ab90395: Manufacturer validation; Connective tissue fibres. Pig skin tissue. WB: Pig skin whole cell lysate; IHC-Fr: Pig Ureter tissue sections. 146 references. Our validation with MG63 homo sopiens osteosarcoma cell cultures.<br>Abcam ab2864: Manufacturer validation; WB: HeLa cell lysate; Rabbit skeletal muscle membrane preparations. IHC-P: Human skeletal muscle tissue; Mouse skeletal muscle and brain tissue; Rat spinal cord tissue. IHC-Fr: Chicken hind brain tissue. ICC/IF: PC12, U251 and HeLa cells. Flow Cyt: C6, Neuro-2A and SH-SY5Ycells.<br>Abcam ab219798: Manufacturer validation; WB: Human skeletal muscle tissue lysate; mouse skeletal muscle tissue lysate; rat skeletal muscle tissue lysate. IHC-P: Mouse skeletal muscle and cerebellum tissue; rat skeletal muscle and cerebellum tissue. IHC-Fr: Mouse skeletal muscle and cerebellum tissue; rat skeletal muscle and cerebellum tissue. ICC/IF: C2C12 cells.

Eukaryotic cell lines

Policy information about cell lines

Cell line source(s)	human Mesenchymal Stromal Cells were obtained from Texas A&M Health Science Center College of Medicine Institute for Regenerative Medicine at Scott & White who isolated and provided the cells through a grant from NCRR of the NIH (Grant #P40RR017447).
Authentication	Cells were not authenticated
Mycoplasma contamination	Cells were tested regularly for mycoplasma contamination
Commonly misidentified lines<br>(See ICLAC register)	*Name any commonly misidentified cell lines used in the study and provide a rationale for their use.*
